# Metastatic site as a predictor of nivolumab efficacy in patients with advanced non-small cell lung cancer: A retrospective multicenter trial

**DOI:** 10.1371/journal.pone.0192227

**Published:** 2018-02-22

**Authors:** Motohiro Tamiya, Akihiro Tamiya, Takako Inoue, Madoka Kimura, Kei Kunimasa, Kenji Nakahama, Yoshihiko Taniguchi, Takayuki Shiroyama, Shun-ichi Isa, Kazumi Nishino, Toru Kumagai, Hidekazu Suzuki, Tomonori Hirashima, Shinji Atagi, Fumio Imamura

**Affiliations:** 1 Osaka International Cancer Institute, Osaka, Japan; 2 National Hospital Organization Kinki-chuo Chest Medical Center, Osaka, Japan; 3 Osaka Prefectural Medical Center for Respiratory and Allergic Diseases, Osaka, Japan; University of South Alabama Mitchell Cancer Institute, UNITED STATES

## Abstract

**Purpose:**

To conduct a retrospective multicenter trial to determine the significance of metastatic site as a predictor of nivolumab efficacy in patients with advanced non-small cell lung cancer.

**Methods:**

This study was conducted across three medical centers in Japan. We retrospectively reviewed all patients who commenced nivolumab treatment at these centers between December 17, 2015 and July 31, 2016. Clinical data were collected, including age, sex, smoking status, Eastern Cooperative Oncology Group performance status, and metastatic site (lymph nodes, liver, brain, bone, lungs [intrapulmonary metastasis], and malignant pleural effusion) at the time of commencing nivolumab treatment. Patients were followed-up until March 31, 2017.

**Results:**

Two hundred and one patients were enrolled. The median age at the time of commencing nivolumab treatment was 68 (range, 27–87) years. One hundred and thirty-five patients were male, 157 patients had a history of smoking, 153 patients had a performance status of 0–1, and 42 patients had squamous cell carcinoma. The median progression-free survival of all patients was 2.5 months. In the univariate analysis, a performance status of ≥2 (hazard ratio [HR]: 1.89, 95.0% confidence interval [CI]: 1.33–2.69; *p* < 0.001) and liver (HR: 2.09, 95.0% CI: 1.35–3.25; *p* < 0.001) and lung (HR: 1.57, 95.0% CI: 1.14–2.16; *p* < 0.01) metastases correlated with a significantly shorter progression-free survival in nivolumab-treated patients. In the multivariate analysis, a performance status of ≥2 (HR: 1.54, 95.0% CI: 1.05–2.25; *p* < 0.05) and liver (HR: 1.90, 95.0% CI: 1.21–2.98; *p* < 0.01) and lung (HR: 1.41, 95.0% CI: 1.00–1.99; *p* < 0.05) metastases were independently correlated with a significantly shorter progression-free survival in nivolumab-treated patients.

**Conclusion:**

Liver and lung metastases and a poor performance status are independent predictors of nivolumab efficacy in patients with advanced non-small cell lung cancer.

## Introduction

Lung cancer is the leading cause of cancer-related deaths worldwide [1]. Until recently, effective treatments have been limited for patients with non-small cell lung cancer (NSCLC) whose disease progresses after first- or second-line chemotherapy. Docetaxel is associated with a longer survival time than best supportive care [2] and erlotinib has been shown to improve overall survival compared with placebo as a second-line chemotherapy for advanced NSCLC [3]. Newer agents (e.g., pemetrexed) have better side effect profiles and have shown non-inferiority to docetaxel. However, these agents have not demonstrated superiority to docetaxel with respect to overall survival when used as a second-line treatment [4]. Recently, combined docetaxel and ramucirumab, a fully humanized immunoglobulin G1 monoclonal antibody that specifically binds with high affinity to the extracellular domain of vascular endothelial growth factor receptor 2, has been shown to improve survival compared with docetaxel alone as a second-line chemotherapy [5]. However, the combination of docetaxel and ramucirumab proved to be more toxic and the benefits were modest. Therefore, novel therapeutic approaches are required.

Programmed cell death 1 (PD-1) is a receptor that is expressed on the surface of activated T cells [6]. It binds to its ligands, programmed death-ligand 1 (PD-L1) and 2, which are commonly expressed in NSCLC. These ligands inhibit T cell activation and promote tumor immune escape [6–8]. Nivolumab, a fully humanized immunoglobulin G4 anti-PD-1 antibody, disrupts PD-1-mediated signaling and restores antitumor immunity [9, 10]. In two key phase III clinical trials [11, 12] of advanced squamous cell (SQ) NSCLC (CheckMate 017) and non-SQ NSCLC (CheckMate 057), nivolumab has shown promising effects and improved overall survival in patients with NSCLC as a second-line or higher treatment. However, the proportion of patients who benefited from nivolumab was <20.0%, even in clinical trials. Therefore, nivolumab may be less effective for unselected patients in a real-world setting. The identification of biomarkers for predicting nivolumab efficacy is crucial.

We conducted a retrospective multicenter trial to determine the significance of clinical factors as predictors of nivolumab efficacy in patients with advanced NSCLC. Particular attention was focused on metastatic site, because the relationship between metastatic site and nivolumab efficacy is unknown.

## Materials and methods

This study was conducted across three medical centers in Japan. The study design was approved by the Institutional Review Board of each participating institution. Research was conducted in accordance with the Declaration of Helsinki and the World Health Organization’s Guidelines for Good Clinical Practice. All study participants have provided informed written consent of receiving nivolumab treatment. The study is registered with the University Hospital Medical Information Network Clinical Trials Registry in Japan (UMIN000025908).

### Patient selection

Two hundred and one patients were enrolled at the Osaka International Cancer Institute, the Osaka Habikino Medical Center, and the National Hospital Organization Kinki-chuo Chest Medical Center between December 17, 2015 (the date nivolumab was approved in Japan) and July 31, 2016. Study participants were consecutively enrolled from patients in routine practice according to the following criteria: nivolumab-treated patients (3.0 mg/kg intravenously every 2 weeks) who had previously been treated for advanced NSCLC (including in one patient who was treated as a first line). Patients were excluded from our analysis if they had received nivolumab treatment as part of a clinical trial or if any additional antineoplastic therapies were administered concurrently.

### Data collection

Clinical data, including age, sex, smoking status, Eastern Cooperative Oncology Group (ECOG) performance status (PS), and metastatic site (lymph nodes (LNs) [thoracic LNs], liver, brain, bone, lung [intrapulmonary metastasis], and malignant pleural effusion [MPE]) at the time of commencing nivolumab treatment, were collected from electronic medical records and pharmacy databases. However, we did not analyze the PD-L1 status. Clinical responses were defined according to the Response Evaluation Criteria in Solid Tumors, version 1.1 [13]. Progression-free survival (PFS) was determined from the date of commencing primary systemic therapy to the date of disease progression or death from any cause. Patients were followed-up until March 31, 2017. All data were analyzed through outsourcing (EP-SOGO Co., Ltd., Tokyo, Japan).

### Statistical analyses

Kaplan-Meier curves were used to evaluate PFS, which was compared using the log-rank test. Median values and 95.0% confidence intervals (CIs) were also reported. Univariate and multivariate analyses were performed using Cox proportional hazards regression models. Only factors with a *p* < 0.05 in the univariate analysis were included in the multivariate analysis. All statistical analyses were conducted using R software, version 2.8.1 (http://R-project.org). A *p* < 0.05 was considered statistically significant and a *p* < 0.10 was considered moderately significant.

## Results

Two hundred and one patients were treated with nivolumab and enrolled in this study (S1 Table). The median follow up time of this study was 12.2 months. At the time of commencing nivolumab treatment, the median age was 68 (range, 27–87) years. One hundred and thirty-five patients were male, 157 patients had a history of smoking, 153 patients had an ECOG PS of 0–1, the median number of previous treatment was 2, 42 patients had SQ carcinoma, and 37 patients had EGFR mutation. Intrapulmonary metastasis, thoracic LNs metastasis, MPE, bone metastasis, brain metastasis, and liver metastasis were observed in 115 (57.2%), 105 (52.2%), 89 (44.3%), 66 (32.8%), 51 (25.4%), and 29 (14.4%) patients, respectively (**Table 1**).

**Table 1:** Patient baseline characteristics.

Characteristic	Patients (n = 201)
Median age (range) (years)	68 (27–87)
Sex (Male/Female)	135/66
Smoking history (smoker / non-smoker)	157/44
Performance status (0/1/2/3/4)	32/121/33/12/3
Median previous treatment (≤2/≥3)	2 (123/78)
Histological types (SCC/ADC/others)	42/142/17
EGFR mutation (positive/negative/unknown)	37/128/36
Thoracic lymph node metastasis	105 (52.2%)
Liver metastasis	29 (14.4%)
Brain metastasis	51 (25.4%)
Bone metastasis	66 (32.8%)
Intrapulmonary metastasis	115 (57.2%)
Malignant pleural effusion	89 (44.3%)
The number of metastatic sites (0/1/2/3/4/5/6)	2 (10/47/70/46/19/6/3)

**Fig 1:**
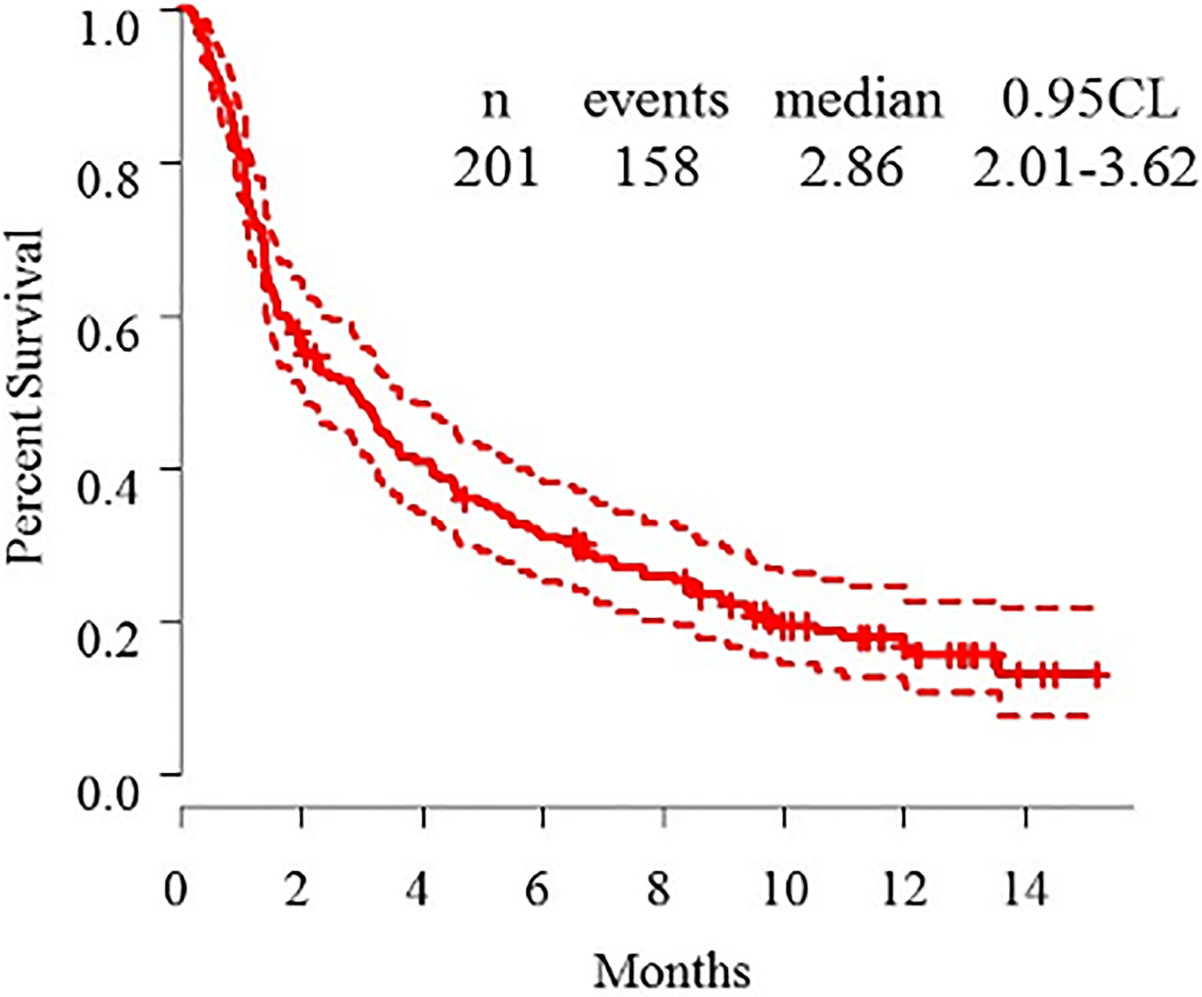
Kaplan-Meier curve of progression-free survival for nivolumab-treated patients with advanced non-small cell lung cancer.

**Fig 2:**
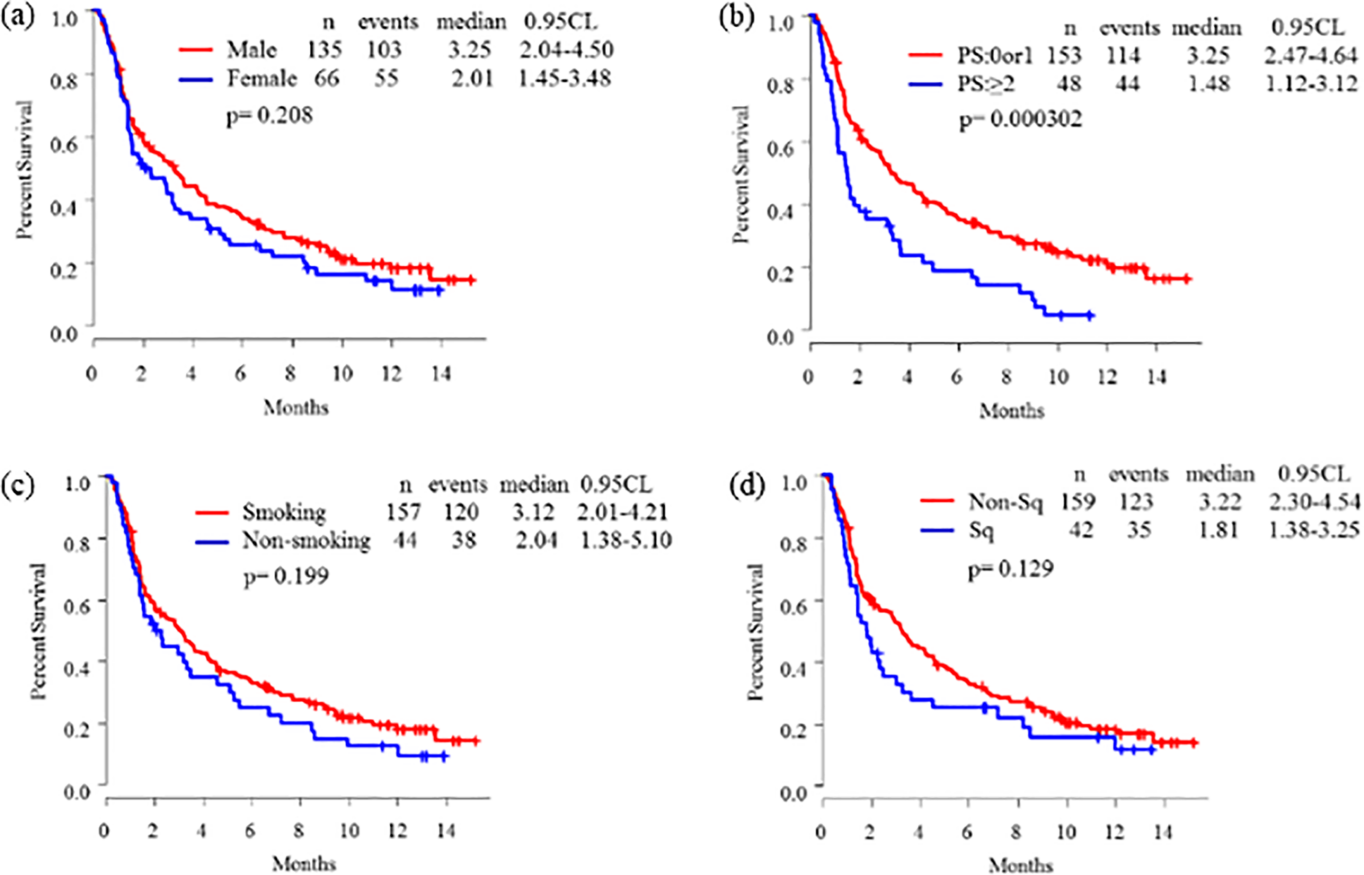
Kaplan-Meier curves of progression-free survival according to (a) sex, (b) Eastern Cooperative Oncology Group performance status (PS), (c) smoking status, and (d) squamous cell (SQ) subtype.

**Fig 3:**
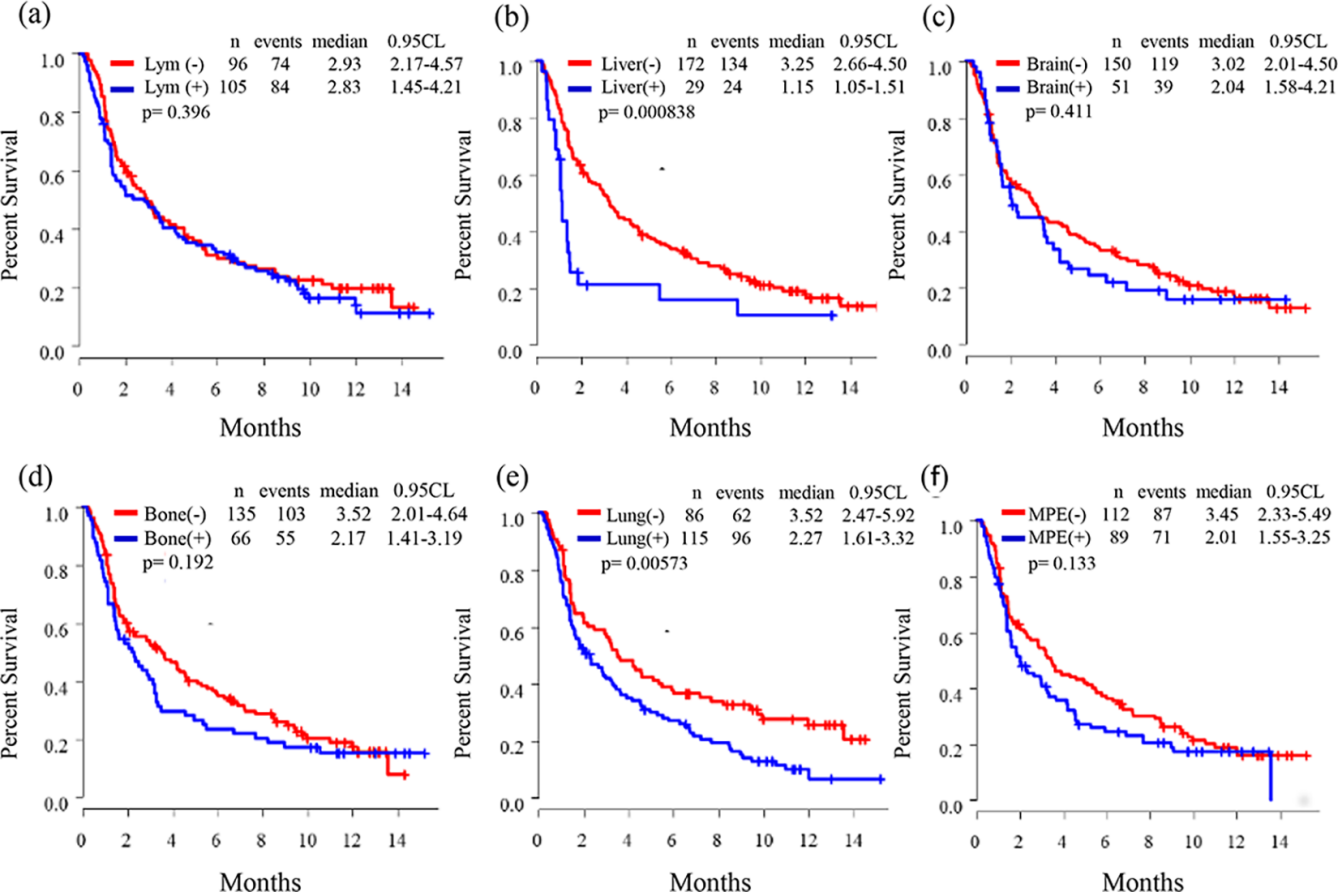
Kaplan-Meier curves of progression-free survival according to metastatic site: (a) thoracic lymph nodes, (b) liver, (c) brain, (d) bone, (e) Lung (intrapulmonary metastasis), and (f) MPE (malignant pleural effusion).

The overall response rate, disease control rate, and progressive disease rates were 15.9%, 51.7%, and 44.8%, respectively. Kaplan-Meier curves of PFS are shown in **Figs 1–3**. The median PFS of all patients was 2.86 (95.0% confidence interval (CI): 2.01–3.62) months (**Fig 1**). There were no difference of median PFS of nivolumab according to sex (**Fig 2A**), smoking status (**Fig 2C**), SQ subtype (**Fig 2D**), thoracic LNs metastasis status (**Fig 3A**), brain metastasis status (**Fig 3C**), bone metastasis status (**Fig 3D**), and MPE status (**Fig 3F**). The median PFS for ECOG PS (0–1 *versus (vs*.*)* ≥2: 3.25 [95.0% CI: 2.47–4.64] *vs*. 1.48 [95.0% CI: 1.12–3.12] months; *p* < 0.001) (**Fig 2B**), liver metastasis status (liver negative v*s*. liver positive: 3.25 [95.0% CI: 2.66–4.50] *vs*. 1.15 [95.0% CI: 1.05–1.51] months; *p* < 0.001) (**Fig 3B**), and intrapulmonary metastasis status (lung negative *vs*. lung positive: 3.52 [95.0% CI: 2.47–5.92] *vs*. 2.27 [95.0% CI: 1.61–3.32] months; *p* < 0.01) (**Fig 3E**) were significantly different.

The univariate Cox proportional hazards regression model of PFS in nivolumab-treated patients with advanced NSCLC was performed. Sex, smoking status, SQ subtype, LN metastasis status, brain metastasis status, bone metastasis status, and MPE status did not correlated with a shorter PFS of nivolumab. ECOG PS (0–1 *vs*. ≥2: HR: 1.89, 95.0% CI: 1.33–2.69; *p* < 0.001), liver metastasis status (liver negative *vs*. liver positive: HR: 2.09, 95.0% CI: 1.35–3.25; *p* < 0.001), and lung metastasis status (lung negative *vs*. lung positive: HR: 1.57, 95.0% CI: 1.14–2.16; *p* < 0.01) correlated with a shorter PFS in nivolumab-treated patients with advanced NSCLC. Furthermore, in the multivariate Cox proportional hazards regression model, an ECOG PS of ≥2 (HR: 1.54, 95.0% CI: 1.05–2.25; *p* < 0.05) and liver (HR: 1.90, 95.0% CI: 1.21–2.98; *p* < 0.01) and lung (HR: 1.41, 95.0% CI: 1.00–1.99; *p* < 0.05) metastases significantly and independently correlated with a shorter PFS in nivolumab-treated patients with advanced NSCLC (**Table 2**).

**Table 2:** Univariate and multivariate analysis according to PFS of Nivolimab.

		Univariate analysis		Multivariate analysis	
Factor		HR(95%CI) Pvalue		HR(95%CI) Pvalue	
SEX	Male	1		NA	
	Female	1.23(0.89–1.71)	0.2079	NA	NA
PS	0 or 1	1		1	
	≥2	1.89(1.33–2.69)	0.0003	1.54(1.05–2.25)	0.0258
Smoking	Smoker	1		NA	
	Non-smoker	1.27(0.88–1.83)	0.2015	NA	NA
Histology	Non-Sq	1		NA	
	Sq	1.34(0.92–1.95)	0.1292	NA	NA
Lym	(-)	1		NA	
	(+)	1.15(0.84–1.57)	0.3911	NA	NA
Liver	(-)	1	1	1	
	(+)	2.09(1.35–3.25)	0.0008	1.90(1.21–2.98)	0.0054
Brain	(-)	1		NA	
	(+)	1.16(0.81–1.67)	0.4139	NA	NA
Bone	(-)	1		NA	
	(+)	1.24(0.90–1.73)	0.1914	NA	NA
Lung	(-)	1		1	
	(+)	1.57(1.14–2.16)	0.0059	1.41(1.00–1.99)	0.0488
MPE	(-)	1		NA	
	(+)	1.27(0.93–1.75)	0.1315	NA	NA
Number	0–2	1	0.0018	NA	
	≥3	1.67(1.21–2.32)		NA	NA

The median number of metastatic sites including in six organs was 2 (0/1/2/3/4/5/6: 10/47/70/46/19/6/3), and the median PFS according to the number of metastatic sites tended to get worse as the number increases (the median PFS of 0 / 1 / 2 / 3 / 4 / 5 / 6: 4.07 / 5.17 / 2.90 / 2.70 / 1.40 / 0.87 / 1.13 months). In univariate analysis, the number of metastatic organ sites among six organs was significantly associated with a shorter PFS of nivolumab. (0–2 *vs*. ≥3: 3.67 [95.0% CI: 2.87–5.60] *vs*. 1.87 [95.0% CI: 1.40–3.00] months; HR: 1.67, 95.0% CI: 1.21–2.32; *p* = 0.002).

## Discussion

Previous studies have shown that among patients receiving third line therapy, those who had never smoking status, those with central nerve system metastases, those with EGFR mutation, and those with poor PS [11,12]. Moreover, the PFS may be shorter in a clinical setting than in a clinical trial. In this study, we particularly focused on between metastatic site and effect of nivolumab.

Nivolumab is less effective in the patients with a poor ECOG PS. They may have a weaker immune system than those with a good ECOG PS, and the lymphocytes of these patients may not function efficiently, even with a high PD-L1 expression status. The contribution of metastatic sites to nivolumab effect is much more complicated. Metastatic spread of cancer to distant organs is the cause of the majority of cancer-related deaths. Previous studies [14, 15] have revealed bone, lungs, liver, brain, and adrenal glands to be the most common sites of extranodal metastasis in patients with NSCLC. In particular, bone and liver metastases are associated with the poorest survival in patients with lung cancer [16]. In contrast, MPE, occurring in approximately 15.0% of patients with NSCLC, influences patient management and quality of life, and is a poor prognostic factor of survival [17, 18]. However, these findings are based on experiences before nivolumab administration in a clinical setting, and the relationship between metastatic site and nivolumab efficacy is unknown.

To the best of our knowledge, this is the first study to analyze the relationship between metastatic site and PFS in a large population of patients with advanced NSCLC who were treated with nivolumab in a real-world setting. Our findings demonstrate that a ECOG PS of ≥2 and liver and lung metastases are independently correlated with a shorter median PFS in nivolumab-treated patients with advanced NSCLC. However, no clear correlation was observed between the metastasis to the other organs and PFS. There are several explanations as to why a difference in the median PFS was observed for each metastatic site in our study.

First is the heterogeneity in PD-L1 expression between primary and metastatic sites. PD-L1 expression status has emerged as a predictive marker of responses to PD-1/PD-L1-directed therapies since the first clinical trials in NSCLC [19]. A high PD-L1 expression is generally associated with a greater effect than a low PD-L1 expression in NSCLC. PD-L1 expression is reported to differ between primary and metastatic sites in patients with melanoma, renal cell carcinoma, and lung cancer [20–25]. Factors that influence PD-L1 expression include tumor hypoxia, a proinflammatory (interferon gamma) microenvironment that promotes cell death and survival [26], and tumor heterogeneity. Tumor heterogeneity influences PD-L1 expression [27]. However, we did not provide PD-L1 expression results for our cohort.

Second is the heterogeneity in genetic profiles between primary and metastatic sites. The difference in the number of somatic mutations among the tumor types and its correlation to the effect of an immune checkpoint inhibitor were reported by Cibulskis *et al*. [28]. Melanoma and NSCLC were associated with the highest numbers of somatic mutations and better response to pembrolizumab. Moreover, impaired mismatch repair was shown to predict the better clinical benefit of pembrolizumab [29]. Kim *et al*. [30] reported on the genetic heterogeneity between primary and metastatic tumors in patients with lung adenocarcinoma. A recent report [31] has highlighted the importance of intratumoral heterogeneity, which drives tumor evolution and drug resistance. Differences in the genetic profiles between lesions are frequently associated with those genes that play an important role in cancer biology. Genetic heterogeneity may influence tumor responses to nivolumab at different metastatic sites.

However, it is difficult to explain with these factors why metastasis to the liver or lung, rather than metastasis to the other organs, worsens the patient's prognosis. The interaction between the tumor and its microenvironment may be considered. A reduced tumor response may be consistent with the immunosuppressive environment of the liver. In a recent report [32], reduced efficacy to liver metastases was shown in patients with melanoma and NSCLC, and liver metastases were associated with decreased marginal cluster of differentiation 8-positive T-cell infiltration. There may be “liver-induced peripheral tolerance”. On the other hand, the reduced efficacy to lung (intrapulmonary) metastases in our study is in contrast to that observed in patients with melanoma [33]. We presumed the reason may be that there is a primary tumor in almost all advanced NSCLC patients and the microenvironment in the lung with a primary tumor may affect the response to nivolumab treatment. In addition, there may be many macrophages surrounding the tumor in the liver and lung. Tumor-associated macrophages express PD-1, and tumor-associated PD-1 expression on macrophages increases over time with increasing disease stages. Tumor-associated PD-1 expression on macrophages inhibits phagocytosis and tumor immunity [34]. Therefore, liver and lung metastasis may correlated with a shorter median PFS time in nivolumab-treated patients with advanced NSCLC.

There are several limitations of this study. First, given the retrospective nature of its design, there is potential for bias. Consequently, all data were analyzed through outsourcing. Confounding effects were addressed by building multivariate models to adjust for confounding factors. Second, PD-L1 expression status could not be included as a potential confounding or interacting variable in our analyses, because of the availability of nivolumab as a second-line or later therapy and the lack of routine PD-L1 testing in NSCLC patients outside of clinical trials in Japan.

## Conclusions

Liver and lung metastases and a poor ECOG PS are independently correlated with a shorter median PFS in nivolumab-treated patients with NSCLC in a real-world setting. Nivolumab-treated patients with NSCLC who have liver and lung metastases and a poor ECOG PS will require careful monitoring.

## Supporting information

S1 Table The minimal data of this analysis. (PDF)
