# Supplementary material for: Metastatic site as a predictor of nivolumab efficacy in patients with advanced non-small cell lung cancer: A retrospective multicenter trial
Source: PLoS One. 2018 Feb 22;13(2):e0192227. doi: 10.1371/journal.pone.0192227 (PMC5823394; doi:10.1371/journal.pone.0192227)
Supplement: S1 Table — (PDF) [file pone.0192227.s001.pdf]

通し番号	event1	pfs	リンパ節転移（Nivo開始時）	肝臓転移（Nivo開始時）	脳転移（Nivo開始時）	骨転移（Nivo開始時）	胸水貯留（Nivo開始時）	肺内転移（Nivo開始時）
1	1	14	有り	有り	無し	有り	有り	有り
2	1	26	無し	有り	無し	無し	無し	有り
3	1	35	無し	無し	無し	無し	無し	無し
4	1	41	無し	無し	無し	無し	有り	有り
5	1	413	無し	無し	無し	無し	有り	無し
6	0	198	有り	無し	無し	無し	有り	有り
7	1	364	有り	無し	無し	無し	無し	無し
8	1	257	有り	無し	無し	無し	無し	有り
9	1	89	無し	無し	無し	有り	有り	無し
10	0	422	有り	無し	無し	有り	無し	無し
11	0	97	無し	無し	無し	無し	有り	有り
12	1	29	Unknown	無し	無し	有り	有り	有り
13	1	163	無し	無し	Unknown	有り	無し	無し
14	1	168	無し	無し	無し	無し	無し	有り
15	1	27	有り	無し	無し	無し	無し	有り
16	1	35	無し	有り	無し	無し	無し	無し
17	1	296	有り	無し	無し	無し	無し	無し
18	1	43	有り	無し	無し	無し	無し	無し
19	1	14	有り	無し	無し	有り	Unknown	有り
20	1	119	無し	無し	有り	無し	無し	有り
21	1	42	無し	無し	無し	無し	無し	無し
22	0	400	無し	有り	無し	無し	無し	無し
23	1	97	無し	無し	無し	有り	無し	有り
24	1	45	無し	無し	無し	無し	無し	有り
25	1	261	無し	無し	無し	無し	無し	有り
26	0	395	無し	無し	無し	有り	無し	有り
27	1	18	無し	無し	無し	有り	有り	有り
28	1	34	有り	無し	無し	有り	無し	無し
29	1	199	有り	無し	無し	無し	無し	有り
30	0	365	有り	無し	有り	無し	無し	無し
31	1	182	無し	無し	無し	無し	無し	無し
32	1	41	有り	有り	無し	有り	有り	無し
33	1	75	無し	無し	無し	有り	無し	無し
34	1	23	無し	無し	無し	有り	有り	有り
35	1	19	有り	無し	有り	有り	無し	有り
36	1	11	無し	無し	無し	有り	無し	有り
37	1	32	無し	有り	無し	無し	無し	有り
38	1	20	無し	無し	有り	有り	無し	無し
39	1	176	有り	無し	無し	無し	無し	有り
40	1	34	無し	無し	無し	無し	無し	無し
41	1	25	無し	無し	無し	無し	有り	有り
42	1	106	有り	無し	有り	有り	無し	無し
43	1	16	有り	有り	無し	有り	有り	無し
44	1	250	有り	無し	無し	有り	無し	無し
45	0	277	有り	無し	無し	無し	無し	無し
46	1	43	有り	無し	無し	無し	無し	無し
47	1	180	有り	無し	無し	無し	有り	無し
48	1	19	有り	無し	無し	無し	無し	無し
49	1	21	有り	無し	有り	無し	有り	有り
50	1	32	有り	有り	有り	有り	有り	有り
51	0	27	有り	無し	有り	無し	無し	有り
52	0	254	有り	無し	無し	無し	有り	無し
53	1	95	無し	無し	無し	有り	無し	無し
54	1	191	有り	無し	有り	無し	無し	有り
55	1	42	有り	無し	無し	有り	有り	有り

56	1	42	有り	無し	無し	無し	有り	有り
57	1	286	有り	無し	無し	無し	無し	無し
58	0	462	有り	無し	無し	有り	無し	有り
59	1	11	無し	無し	無し	無し	有り	無し
60	1	209	有り	無し	無し	無し	有り	有り
61	1	26	有り	有り	有り	有り	無し	有り
62	0	68	無し	有り	無し	無し	有り	有り
63	1	62	無し	無し	有り	有り	有り	有り
64	1	26	有り	無し	有り	有り	有り	有り
65	1	27	無し	無し	有り	有り	無し	有り
66	1	14	有り	有り	有り	有り	有り	無し
67	1	41	有り	有り	有り	無し	有り	有り
68	0	441	無し	無し	無し	有り	無し	無し
69	1	233	有り	無し	無し	有り	有り	無し
70	1	97	無し	無し	無し	有り	無し	無し
71	0	434	無し	無し	有り	有り	無し	無し
72	1	55	無し	無し	無し	無し	有り	有り
73	1	35	無し	有り	無し	無し	無し	無し
74	1	81	無し	無し	無し	有り	有り	有り
75	1	273	有り	有り	有り	有り	有り	有り
76	1	128	有り	無し	有り	無し	有り	無し
77	1	138	無し	無し	無し	無し	有り	無し
78	1	15	無し	無し	無し	無し	有り	有り
79	1	167	無し	有り	有り	有り	無し	無し
80	1	366	有り	無し	無し	無し	無し	有り
81	1	150	無し	無し	無し	有り	無し	有り
82	0	434	有り	無し	無し	無し	無し	無し
83	1	30	有り	無し	有り	無し	無し	有り
84	1	54	有り	無し	無し	無し	無し	有り
85	1	60	有り	無し	有り	無し	無し	無し
86	1	28	無し	無し	有り	有り	無し	有り
87	1	7	有り	無し	無し	有り	有り	有り
88	1	29	無し	無し	無し	無し	有り	有り
89	1	110	有り	無し	有り	無し	無し	有り
90	1	38	無し	無し	無し	無し	無し	有り
91	0	59	無し	無し	有り	無し	無し	有り
92	1	10	有り	無し	有り	無し	無し	有り
93	1	11	有り	無し	無し	無し	有り	有り
94	1	29	有り	有り	有り	無し	無し	有り
95	1	34	無し	無し	無し	有り	有り	無し
96	1	11	有り	無し	無し	無し	有り	無し
97	1	48	無し	無し	有り	有り	有り	有り
98	1	50	無し	無し	有り	無し	無し	有り
99	1	32	無し	無し	無し	無し	有り	有り
100	0	31	有り	有り	有り	無し	無し	無し
101	1	61	有り	無し	無し	無し	有り	有り
102	1	87	無し	無し	無し	有り	無し	有り
103	1	218	無し	無し	有り	無し	無し	無し
104	1	131	有り	無し	無し	無し	無し	無し
105	1	38	有り	無し	無し	無し	有り	有り
106	1	126	有り	無し	無し	無し	有り	有り
107	0	393	無し	無し	無し	無し	無し	無し
108	1	155	有り	無し	無し	無し	無し	無し
109	1	276	無し	無し	無し	無し	有り	有り
110	0	387	無し	無し	無し	無し	有り	無し
111	1	205	有り	無し	無し	無し	無し	有り
112	1	334	無し	無し	無し	無し	無し	有り
113	1	49	無し	無し	有り	無し	無し	有り
114	1	204	無し	無し	無し	有り	無し	有り

115	0	143	無し	無し	有り	無し	有り	有り
116	0	63	無し	無し	有り	無し	有り	有り
117	0	30	有り	無し	無し	無し	有り	有り
118	0	372	無し	無し	無し	無し	無し	無し
119	1	43	無し	無し	無し	有り	無し	無し
120	1	49	有り	無し	無し	有り	有り	無し
121	0	354	無し	無し	無し	無し	有り	有り
122	1	321	無し	無し	無し	有り	無し	有り
123	0	352	無し	無し	無し	有り	無し	有り
124	1	107	有り	無し	有り	無し	無し	無し
125	0	203	有り	無し	無し	無し	無し	無し
126	0	343	有り	無し	無し	無し	有り	有り
127	1	47	無し	無し	無し	無し	有り	無し
128	0	346	無し	無し	有り	有り	無し	有り
129	0	371	有り	無し	無し	無し	有り	無し
130	1	90	有り	無し	無し	無し	有り	有り
131	0	343	無し	無し	無し	有り	有り	無し
132	1	139	無し	無し	無し	有り	有り	有り
133	1	17	有り	無し	無し	無し	有り	無し
134	1	259	無し	無し	無し	無し	無し	有り
135	1	15	有り	有り	無し	無し	無し	無し
136	1	13	有り	有り	無し	有り	無し	有り
137	1	48	有り	無し	有り	無し	有り	無し
138	1	33	無し	無し	有り	無し	無し	有り
139	1	57	無し	有り	無し	有り	有り	有り
140	1	34	有り	有り	有り	有り	有り	有り
141	0	315	有り	無し	無し	有り	有り	有り
142	0	308	無し	無し	有り	有り	有り	有り
143	0	99	無し	無し	無し	無し	無し	有り
144	1	111	無し	無し	無し	無し	有り	有り
145	1	71	有り	無し	無し	無し	有り	有り
146	1	70	無し	無し	無し	有り	無し	有り
147	1	61	無し	無し	無し	無し	無し	無し
148	1	98	無し	無し	無し	無し	有り	無し
149	1	128	無し	無し	有り	無し	有り	無し
150	1	49	無し	無し	無し	無し	有り	有り
151	1	105	有り	無し	有り	無し	無し	有り
152	0	297	有り	無し	有り	無し	有り	有り
153	1	86	無し	無し	無し	無し	無し	有り
154	0	295	有り	無し	無し	無し	有り	無し
155	0	289	無し	無し	無し	無し	無し	無し
156	1	141	有り	無し	無し	無し	有り	有り
157	1	33	有り	有り	無し	無し	無し	無し
158	1	24	有り	有り	無し	無し	無し	有り
159	1	86	有り	無し	無し	無し	無し	有り
160	1	7	有り	有り	無し	無し	有り	有り
161	0	200	有り	無し	有り	無し	無し	無し
162	0	262	有り	無し	有り	無し	有り	無し
163	1	110	有り	無し	無し	無し	無し	無し
164	0	262	有り	無し	無し	無し	無し	無し
165	1	61	有り	無し	有り	無し	有り	無し
166	0	199	無し	無し	無し	無し	無し	無し
167	1	27	有り	無し	無し	無し	無し	無し
168	1	140	無し	無し	有り	無し	無し	無し
169	1	92	有り	無し	無し	無し	有り	無し
170	1	42	有り	無し	無し	有り	有り	無し
171	1	42	有り	無し	有り	無し	有り	有り
172	0	56	有り	有り	無し	有り	無し	有り
173	1	44	有り	有り	有り	有り	無し	有り

174	1	37	無し	無し	無し	無し	有り	有り
175	1	219	有り	無し	無し	無し	無し	有り
176	1	99	無し	無し	無し	有り	無し	無し
177	0	409	無し	無し	無し	無し	有り	無し
178	1	5	有り	無し	無し	有り	無し	有り
179	1	66	無し	無し	無し	有り	無し	無し
180	1	42	有り	有り	無し	無し	無し	有り
181	0	401	無し	有り	無し	無し	無し	無し
182	1	71	無し	無し	有り	有り	無し	有り
183	1	160	無し	無し	無し	無し	有り	有り
184	1	233	無し	無し	無し	無し	有り	有り
185	1	22	有り	無し	無し	無し	有り	有り
186	1	69	無し	無し	有り	無し	有り	有り
187	1	34	有り	無し	無し	有り	無し	無し
188	1	99	有り	無し	無し	有り	有り	有り
189	1	16	無し	無し	無し	無し	有り	有り
190	1	288	有り	無し	無し	無し	無し	有り
191	1	302	有り	無し	無し	無し	無し	無し
192	1	101	有り	無し	無し	有り	有り	有り
193	1	28	無し	無し	無し	無し	無し	無し
194	1	42	有り	無し	無し	無し	有り	無し
195	0	303	有り	無し	無し	無し	無し	無し
196	1	137	有り	無し	無し	無し	有り	有り
197	1	46	無し	有り	有り	有り	無し	有り
198	1	37	無し	無し	無し	無し	有り	無し
199	1	56	有り	無し	無し	無し	有り	有り
200	1	25	有り	無し	無し	無し	有り	無し
201	1	33	有り	無し	無し	無し	無し	有り

PS（Nivo開始時）	性別	年齢（Nivo開始時）	組織型	喫煙歴	EGFR変異（診断時）
2	男	27	扁平上皮	never	negative
1	男	68	扁平上皮	former	negative
0	男	58	腺癌	former	exon19 del
0	男	71	腺癌	former	negative
1	男	67	腺癌	former	negative
1	女	61	腺癌	former	negative
0	男	70	扁平上皮	former	negative
2	女	70	腺癌	never	exon19 del
1	女	67	その他	former	negative
0	女	80	腺癌	never	negative
2	男	73	腺癌	former	negative
1	男	66	腺癌	former	その他Double m+
1	男	69	腺癌	former	exon19 del
1	男	57	腺癌	former	negative
0	男	65	腺癌	former	negative
0	男	49	扁平上皮	former	Unknown
0	男	72	腺癌	current	negative
0	男	61	扁平上皮	former	negative
1	女	64	腺癌	never	negative
1	女	67	腺癌	former	negative
1	男	72	大細胞癌	former	negative
0	男	74	腺癌	former	negative
0	女	72	腺癌	former	exon19 del
1	男	80	腺癌	former	negative
1	女	48	腺癌	never	negative
0	女	54	腺癌	never	negative
2	女	68	腺癌	never	negative
2	男	60	扁平上皮	current	negative
2	男	53	腺癌	current	negative
1	男	71	腺癌	former	negative
0	男	59	腺癌	current	negative
1	女	75	腺癌	never	negative
1	男	62	扁平上皮	current	negative
1	男	87	腺癌	former	negative
0	男	74	腺癌	current	negative
1	男	71	扁平上皮	former	negative
1	男	51	腺癌	former	Unknown
4	女	72	腺癌	never	negative
1	男	69	腺癌	former	negative
0	女	61	腺癌	never	exon19 del
1	男	65	腺癌	never	negative
1	女	57	腺癌	never	exon19 del
3	女	45	腺癌	former	negative
0	男	74	扁平上皮	former	negative
0	男	60	腺癌	former	negative
2	男	74	扁平上皮	former	negative
1	男	67	腺癌	former	negative
1	男	67	扁平上皮	former	negative
1	男	72	腺がん	former	negative
1	男	74	扁平上皮	current	negative
1	男	56	腺扁平上	current	negative
1	男	67	腺がん	former	negative
3	男	64	腺がん	current	negative
1	男	65	腺癌	former	negative
1	女	81	腺癌	former	negative

1	女	63	腺癌	never	exon19 del
1	男	45	腺癌	former	negative
1	男	55	腺癌	current	negative
2	男	67	腺癌	former	negative
1	男	61	その他	former	negative
3	女	59	腺扁平上[illegible]	current	negative
2	男	75	扁平上皮[illegible]	current	Unknown
1	男	67	腺癌	never	negative
2	男	72	腺癌	former	exon21 L858R
2	女	54	腺癌	never	exon19 del
1	男	66	腺癌	current	negative
1	男	68	その他	former	Unknown
1	男	66	腺癌	former	negative
1	男	59	腺癌	former	negative
0	女	61	腺癌	never	exon21 L858R
1	男	65	腺癌	former	exon19 del
1	男	63	腺癌	former	negative
2	男	64	腺癌	former	negative
1	男	71	腺癌	former	exon19 del
2	女	57	腺癌	former	negative
1	男	67	腺癌	current	negative
1	男	68	腺癌	current	negative
1	女	64	腺癌	never	exon19 del
1	女	85	腺癌	never	exon19 del
1	女	78	腺癌	never	exon21 L858R
2	男	79	腺扁平上[illegible]	former	Unknown
1	男	79	腺癌	former	negative
3	男	56	扁平上皮[illegible]	former	Unknown
2	男	65	扁平上皮[illegible]	former	Unknown
1	男	52	扁平上皮[illegible]	current	Unknown
2	女	62	腺癌	never	Unknown
1	女	70	腺癌	never	exon19+exon18
1	男	77	腺癌	former	negative
2	男	74	扁平上皮[illegible]	former	Unknown
1	男	74	腺癌	former	negative
0	女	61	腺癌	never	negative
1	女	75	腺癌	former	negative
1	男	77	扁平上皮[illegible]	former	negative
2	女	65	腺癌	never	exon19 del
4	男	82	その他	former	exon18
2	男	68	腺癌	former	negative
1	女	63	腺癌	never	exon21 L858R
1	男	66	その他	former	Unknown
1	女	68	腺癌	current	negative
1	男	70	腺癌	former	exon19 del
1	男	75	腺癌	current	exon18
1	女	79	腺癌	former	negative
0	男	66	扁平上皮[illegible]	current	Unknown
0	男	73	腺癌	former	negative
0	男	67	腺癌	current	negative
1	男	84	腺癌	former	negative
1	女	66	腺癌	former	negative
1	女	62	腺癌	never	exon19 del
2	男	78	腺癌	former	negative
1	男	46	扁平上皮[illegible]	current	Unknown
2	男	81	腺癌	former	negative
0	女	66	腺癌	former	negative
2	男	79	腺癌	former	negative
1	女	66	腺癌	never	exon19del

1	女	64	腺癌	former	exon21 L858R
1	女	73	腺癌	never	negative
1	男	71	腺癌	former	negative
0	男	53	扁平上皮	former	Unknown
1	男	57	扁平上皮	current	Unknown
1	男	83	腺癌	former	negative
0	男	71	腺癌	former	negative
1	男	55	腺癌	former	negative
1	男	72	腺癌	former	negative
1	男	65	腺癌	former	Unknown
1	男	76	扁平上皮	former	Unknown
1	女	73	扁平上皮	current	Unknown
2	女	83	腺癌	never	negative
1	女	67	腺癌	never	exon21 L858R
1	男	59	腺癌	former	Unknown
1	女	83	腺癌	never	negative
2	男	80	腺癌	former	negative
2	男	63	腺癌	former	negative
1	女	75	扁平上皮	current	Unknown
1	男	75	扁平上皮	former	Unknown
3	男	51	扁平上皮	current	Unknown
3	男	64	その他	former	exon18
1	女	57	扁平上皮	former	exon20 S768I
3	女	66	腺癌	former	exon19 del
1	女	72	腺癌	never	negative
3	男	66	腺癌	current	negative
1	男	77	腺癌	former	negative
2	男	76	腺癌	former	negative
1	男	84	腺癌	former	negative
2	男	79	その他	current	exon18
1	男	71	扁平上皮	former	Unknown
1	女	70	腺癌	never	negative
1	女	70	その他	current	negative
1	男	76	腺癌	former	negative
1	男	79	腺癌	former	negative
2	男	83	腺癌	former	negative
1	男	72	その他	former	negative
1	男	63	腺癌	former	negative
0	男	61	腺癌	former	negative
0	男	71	腺癌	former	negative
0	男	76	その他	former	Unknown
1	女	58	腺癌	former	exon19 del
1	男	72	腺癌	current	Unknown
1	男	67	扁平上皮	current	exon19 del
1	男	65	その他	current	Unknown
1	男	83	腺癌	current	negative
1	男	71	扁平上皮	current	negative
1	男	55	その他	current	negative
1	男	69	腺癌	current	negative
1	女	76	腺癌	former	negative
4	男	71	扁平上皮	former	negative
1	男	54	腺癌	former	negative
1	男	73	扁平上皮	former	Unknown
1	女	69	腺癌	never	exon19 del
1	男	75	扁平上皮	former	Unknown
1	F	76	ad	0	neg
1	M	70	ad	25*51	neg
1	M	70	ad	44*40	neg
2	F	61	ad	20*43	neg

1	F	67	ad		0	neg
1	F	79	ad		0	neg
1	F	62	ad	15*15		neg
1	M	78	Sq	40*30		未
3	M	67	ad	20*50		neg
1	M	71	ad	20*43		L858R
2	F	55	ad	15*33		neg
0	F	63	ad		0	neg
1	F	61	ad		0	neg
1	F	75	ad		0	L858R
1	M	70	ad	40*42		neg
1	F	76	ad		0	L858R
3	M	72	Sq	20*50		未
1	M	71	ad	40*37		neg
2	M	72	Sq	40*50		未
3	M	67	sarcoma	15*37		未
2	M	75	ad	17*40		neg
1	M	64	ad		0	neg
3	M	70	ad		0	neg
1	F	68	Sq	7*45		未
1	F	69	Sq＋sm	30*48		neg
1	M	59	ad	20*40		neg
0	M	61	Sq	25*43		未
2	F	45	ad		0	neg
0	M	74	ad	15*32		neg
0	F	66	Sq	20*42		未
0	M	57	Sq	20*32		未
1	F	68	ad		0	Exon19del
